# Tilt testing remains a valuable asset

**DOI:** 10.1093/eurheartj/ehab084

**Published:** 2021-02-24

**Authors:** Richard Sutton, Artur Fedorowski, Brian Olshansky, J Gert van Dijk, Haruhiko Abe, Michele Brignole, Frederik de Lange, Rose Anne Kenny, Phang Boon Lim, Angel Moya, Stuart D Rosen, Vincenzo Russo, Julian M Stewart, Roland D Thijs, David G Benditt

**Affiliations:** Department of Cardiology, Imperial College, London, UK; Department of Cardiology, Skåne University Hospital and Lund University, Malmö, Sweden; Department of Cardiology, University of Iowa, Iowa City, IA, USA; Department of Neurology, Leiden University Medical Centre, The Netherlands; Department of Heart Rhythm Management, University of Occupational and Environmental Health, Kitakyushu, Japan; Istituto Auxologico Italiano, Faint & Fall Programme, Ospedale San Luca, Milano, Italy; Department of Clinical and Experimental Cardiology, Amsterdam Cardiovascular Sciences, Amsterdam University Medical Centre, University of Amsterdam, Heart Centre, Amsterdam, The Netherlands; School of Medicine, Trinity College, Dublin, Ireland; Department of Cardiology, Hammersmith Hospital, Imperial College, London, UK; Department of Cardiology, Dexeus University Hospital, Barcelona, Spain; National Heart & Lung Institute, Royal Brompton Hospital, Imperial College, London, UK; Department of Translational Sciences, University of Campania, Naples, Italy; Department of Pediatrics, New York Medical College, Valhalla, NY, USA; Department of Neurology, Leiden University Medical Centre, The Netherlands; Cardiac Arrhythmia Center, Cardiovascular Division, University of Minnesota, Minneapolis, MN, USA

**Keywords:** Tilt-table test, Vasovagal syncope, Syncope, Orthostatic hypotension, Postural orthostatic tachycardia syndrome, Psychogenic pseudosyncope, Active stand, ECG-loop recorders

## Abstract

Head-up tilt test (TT) has been used for >50 years to study heart rate/blood pressure adaptation to positional changes, to model responses to haemorrhage, to assess orthostatic hypotension, and to evaluate haemodynamic and neuroendocrine responses in congestive heart failure, autonomic dysfunction, and hypertension. During these studies, some subjects experienced syncope due to vasovagal reflex. As a result, tilt testing was incorporated into clinical assessment of syncope when the origin was unknown. Subsequently, clinical experience supports the diagnostic value of TT. This is highlighted in evidence-based professional practice guidelines, which provide advice for TT methodology and interpretation, while concurrently identifying its limitations. Thus, TT remains a valuable clinical asset, one that has added importantly to the appreciation of pathophysiology of syncope/collapse and, thereby, has improved care of syncopal patients.

## Graphical Abstract

**Figure:**
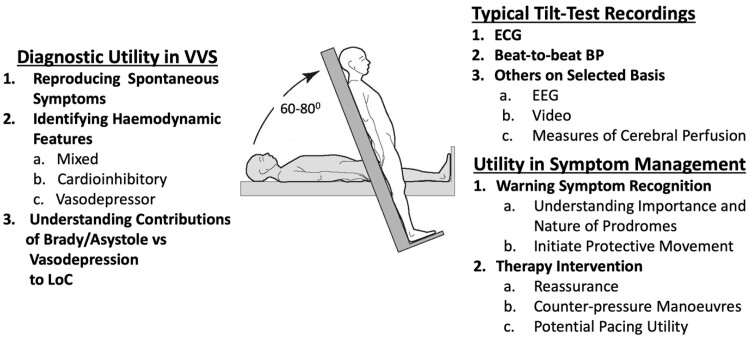

## Introduction

Head-up tilt test (TT) has been used for more than half a century by physiologists and physicians to study heart rate and blood pressure adaptation to positional changes, to model responses to haemorrhage, to assess characteristics of orthostatic hypotension (OH), and to evaluate haemodynamic and neuroendocrine responses in congestive heart failure, autonomic dysfunction, and hypertension. During these studies, some subjects experienced total or near-total transient loss of consciousness (TLOC) due to hypotension induced by TT (often accompanied by bradycardia/asystole).^1–4^ Consequently, beginning in late 1980s, TT was incorporated into clinical assessment of syncope of unknown origin^1^ as a method of triggering the vasovagal reflex in susceptible individuals by exposing them to controlled orthostatic challenge in a safe, monitored, clinical laboratory environment.^1^ ^,^ ^5–8^ However, the clinical utility of TT has been criticised most recently by Kulkarni *et al.*,^9^ who promoted the less well-studied active stand test largely based on the presumption of lesser expense and, perhaps, greater convenience. In this review, while acknowledging TT limitations, we aim to offer counterpoint to the views of Kulkarni *et al.* ^9^ by emphasizing both TT's well-documented clinical value and recommendations by multiple practice guidelines (*Table 1*).

**Table 1:** Pros and cons of tilt testing, active standing, and implantable loop recorders/insertable cardiac monitors

Diagnostic	1. TT helps assess susceptibility to VVS and/or OH in a controlled, safe environment AS is useful only in immediate and classical OH ILR/ICM offers delayed diagnosis, without BP
	2. TT identifies patients with asystole who may require cardiac pacing based on temporal relationship of bradycardia/hypotension^12^ ^,^ ^39^ AS has no value ILR/ICM may identify asystole during spontaneous attacks
	3. TT helps determine similarity of induced to spontaneous clinical symptoms AS is unlikely to be tolerable long enough to obtain this information ILR/ICM is very useful in recording arrhythmia/muscle artefacts during spontaneous attacks
	4. TT identifies syncope mimics (PPS/PNES/ictal asystole) AS has no value ILR/ICM may show normal rhythm during syncope but no BP
	5. TT offers safe, accessible means to study pathophysiology of syncope using, if necessary, EEG, cerebral perfusion assessment AS has no value ILR/ICM shows only arrhythmias
Educational	1. TT helps in educating patients regarding identifying prodrome prompting preventive measures Reassurance by diagnosis of observed attack promoting confidence in recommended therapy AS may have value in teaching patients counterpressure manoeuvres, especially with displayed beat-to-beat BP ILR/ICM has no value
	2. TT provides insight into syncope pathophysiology and its relation to treatment options - Better understanding of the timing relationship between vasodepression, TLOC, and cardioinhibition^39^ - TLOC occurring before cardioinhibition implies strong reluctance towards pacing therapy^39^ AS has no role ILR/ICM may confirm arrhythmic component but without BP
Therapy selection-pacing	TT shows asystole occurring after TLOC permitting avoidance of unnecessary pacing^39^ TT shows asystole before or coincides with hypotension points to symptomatic improvement with pacing^12^ ^,^ ^43^ AS has no role ILR/ICM shows asystole in spontaneous attack but incurs diagnostic delay awaiting further syncope and yields no BP
Conditions other than VVS	TT is optimal in OH (especially delayed OH), PPS/PNES/ictal asystole. TT is preferred for POTS^10^ ^,^ ^11^ AS cannot replace TT as standing unsupported for sufficient time is intolerable plus need for beat-to-beat BP, ECG in all and EEG in some to achieve clear result AS may be adequate for POTS ILR/ICM cannot offer definitive diagnosis except arrhythmia
Major limitations of TT, AS, and ILR	Tilt-induced reflex may not be identical to spontaneous attacks with bradyarrhythmias being more frequent in spontaneous attacks TT is time-consuming, requires training for adequate interpretation, examination protocols differ with results not necessarily comparable TT lab requires beat-to-beat monitor for optimal diagnostic accuracy TT has false positives that should be identified as not reproducing their attack, prompting other tests; also false negatives over which history takes precedence AS must be interrupted as soon as patients report prodromes or cannot stand without support leading to incomplete recording of events AS cannot be used to study pathophysiology of cardiovascular dysautonomia/syncope when prolonged orthostatic challenge is required AS invokes leg-muscle pump ILR/ICM involves diagnostic delay, recurrence of syncope required with trauma risk ILR/ICM cannot record BP during syncope ILR/ICM is minimally invasive/costly in hardware and monitoring

AS, active standing; BP, blood pressure; EEG, electroencephalography; ECG, electrocardiogram; ICM, insertable cardiac monitor; ILR, implantable loop recorder; OH, orthostatic hypotension; PNES, psychogenic non-epileptic seizures; POTS, postural orthostatic tachycardia syndrome; PPS, psychogenic pseudosyncope; TLOC, transient loss of consciousness; TT, tilt test; VVS, vasovagal syncope.

## Current status of tilt testing

A positive TT has diagnostic value in syncope/collapse when the history does not provide a conclusive explanation for symptoms.^10^ ^,^ ^11^ If the history yields a clear diagnosis, TT is not required; nonetheless, TT may provide important patient education and reassurance, together with pathophysiological evidence of the underlying mechanisms, critical for the selection of appropriate therapy.^12^

The methodology and interpretation of TT results have evolved since it was introduced into clinical practice.^1^ Initially, prolonged TTs, up to 2 h at angles 40–60°, were used to trigger vasovagal events in susceptible individuals. Subsequently, test duration was shortened, head-up angle was defined as 60–80°, and other interventions were added to improve test sensitivity.^13–21^ These interventions included administration of drugs (e.g. isoproterenol, nitroglycerine, serotonin agonists) alone or in conjunction with physical manoeuvres, such as carotid sinus massage. Several of these provocative measures improved TT sensitivity and remain in use; however, their addition may reduce specificity.

Regarding TT for evaluating the syncope of unknown origin, Forleo *et al.* ^22^ reported a meta-analysis of 55 studies incorporating patients with unexplained syncope and asymptomatic controls without history of syncope. The authors excluded studies with <10 patients and procedures with tilt angulation <60° or >80°; the evaluation thereby comprised 4361 patients with syncope (aged 41 ± 17 years) and 1791 controls (aged 39 ± 17 years). The summary receiver-operating curve demonstrated good overall ability to differentiate symptomatic patients from asymptomatic controls with an area under the curve of 0.84 [95% confidence interval (CI) 0.81–0.87]. As expected, pharmacological protocols enhanced sensitivity but reduced specificity. Tilt protocols that included nitroglycerine provocation had the highest diagnostic odds ratio (14.40; 95% CI 11.50–18.05) and greatest sensitivity (66%; 95% CI 60–72%).

Given the preponderance of evidence, and working independently (a few members of each group provided reviews of the other document), the European Society of Cardiology (ESC)^10^ and the American College of Cardiology/American Heart Association/Heart Rhythm Society collaboration^11^ arrived at similar and closely coherent recommendations for TT in unexplained syncope after initial clinical assessment [i.e. detailed history and basic examination including electrocardiogram (ECG) and orthostatic blood pressure measurement], agreeing on a class IIA indication. Furthermore, both groups proposed that when an autonomic disturbance was deemed likely, TT (with additional cardiovascular autonomic assessment, if appropriate) should be a preferred component of the diagnostic strategy.

Recent criticisms by Kulkarni *et al.* ^9^ suggest that, like any diagnostic test, TT can be inappropriately applied. Nevertheless, extensive experience, as well as evidence-based practice guideline recommendations, provides clear direction for its appropriate application and indicates when so TT is an important, effective diagnostic tool. Use of other orthostatic stressors might be contemplated (e.g. active standing, squat-stand test), but these have not undergone the scrutiny as potential clinical tools to the degree that has TT, excepting evaluation of initial and classical OH and postural orthostatic tachycardia syndrome where active standing is well recognized, supported by evidence and, thus, by guidelines.^10^ ^,^ ^11^

## Syncope and tilt testing

Tilt-table testing was introduced into clinical evaluation of TLOC of unknown aetiology to assess susceptibility to vasovagal reflex. Such testing is unnecessary for diagnosis if medical history is classical and diagnostic of reflex syncope. However, that is often not the case, especially in older patients in whom the history may be inadequate due in part to retrograde amnesia in these older fainters.^23^

TLOC has four features that can be derived from history taking: (i) tendency to fall as expression of loss of motor control; (ii) amnesia for duration of TLOC; (iii) abnormal responses to speech/touch; and (iv) short duration (<5 min).

TLOC differential diagnosis includes: (i) concussion, (ii) syncope; (iii) epileptic seizures; (iv) psychogenic spells resembling syncope [psychogenic pseudosyncope (PPS)] or seizures [psychogenic non-epileptic seizures (PNES)]; and (v) intoxication/metabolic disturbance (strictly not TLOC as duration is >5 min).

Distinction between these diagnostic entities by careful medical history including eyewitness reports is often but not always possible.

In some patients with recurrent apparent syncope, in whom previous attempts have failed to establish a diagnosis, TT is the best next step and guidelines support this strategy.^10^ ^,^ ^11^ For example, if PPS, PNES, or mechanical falls due to orthostatic intolerance are possible explanations, observations during TT are likely to be diagnostic. Concomitant use of electroencephalography (EEG) is readily added to TT and is considered essential in PPS/PNES.^24^ In OH, TT allows safe prolonged blood pressure assessment without risk of falls and injury such as might occur during active stand or squat-stand tests. However, TT is less effective than active standing for documenting immediate OH, where the latter is recommended.^10^ ^,^ ^11^

**Figure 1:**
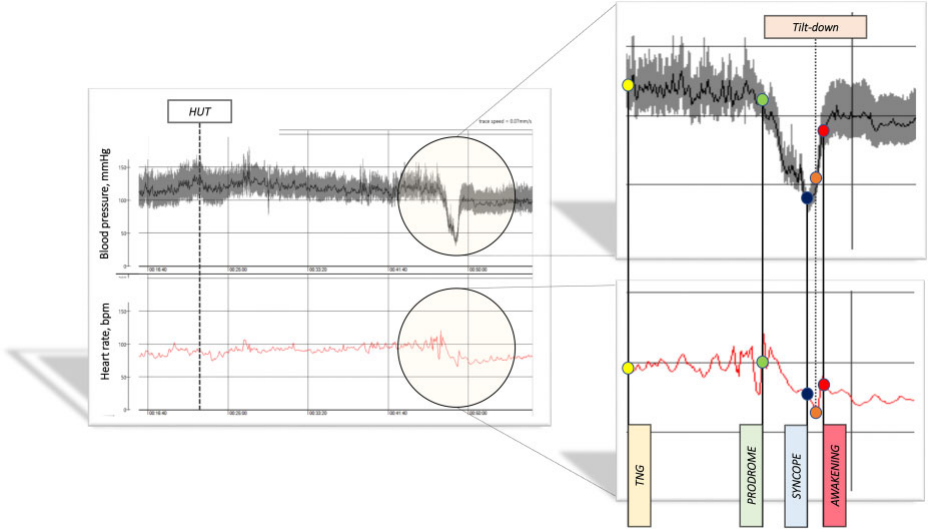
Courtesy of Artur Fedorowski MD and Fabrizio Ricci MD (Malmo, Sweden) depicting blood pressure (upper traces) and heart rate (lower trace) during a head-up tilt test-induced mixed collapse pattern of vasovagal faint. In this case, nitroglycerin was administered sublingually after 20 min of passive upright posture proved non-diagnostic. The expanded images illustrate the collapse using the same format beginning with administration of nitroglycerin; events, prodrome, syncope, tilt-down and awakening are labelled. Time base is shown between the left two panels in minutes. The scales are blood pressure 0–150 mmHg and heart rate 0–150 bpm.

Multiple observations suggest that reported syncope/collapse associated with positive TT is comparable with spontaneous vasovagal syncope (VVS), although it should be accepted that tilt-induced syncope is not identical to the spontaneous attack. For example, the bradyarrhythmias seen on implantable loop recorders (ILR) are more prominent than during TT.^6^ However, VVS diagnosis from TT is based on the patient-recognizing symptom reproduction (*Figure 1*). Thus, TT can play an important role in VVS diagnosis but much less in therapy selection.^5^ ^,^ ^10^ ^,^ ^11^ ^,^ ^25^

The Fainting Assessment Study (FAST),^25^ the clinical study of Wieling *et al.*,^26^ and the review of Sutton *et al.* ^27^ reported a diagnostic yield of ∼60% achieved by hospital physicians. The review later showed the diagnostic yield rises from 60% to 70% by hospital physicians following ESC guidelines to 85% in syncope units where TT, albeit not applied in all cases, and expert history taking and interpretation are available.^25–27^

Finally, clinicians caring for syncope/collapse victims realize that in patients of all ages, recurrent unexplained syncope/collapse may provoke considerable anxiety in those affected and their families. In the case of faints due to VVS/OH/PPS, the patient's understanding that the physician or highly trained assistant^28^ has witnessed their attack and, thereby, has a firm diagnosis is greatly reassuring. TT offers this opportunity.

## Tilt-test methodology

Detailed discussion of TT protocols is provided in several practice guidelines and consensus reports.^8^ ^,^ ^10^ ^,^ ^11^ Failure to follow protocols, especially for induction of syncope, will lead to misinterpretation. In addition, the European Heart Rhythm Association has recommended staffing requirements for performing tilt-table testing including use of highly trained personnel other than physicians.^28^

The 2018 ESC syncope guidelines^10^ give tilt-table testing a IIb indication (level of evidence C) to discriminate convulsive syncope from epilepsy. Misdiagnosis of epilepsy as syncope is a more frequently recognized problem and tilt-table testing has been shown to be helpful in this regard.^29^ The addition of EEG monitoring to assist in making this distinction has proved particularly valuable and may readily be added to TT.^30^ ^,^ ^31^

Increasingly, laboratories that undertake TT are encouraged to include active stand testing in assessing patients.^9^ ^,^ ^10^ However, active standing should not be confused with TT. While both introduce orthostatic stress, there are important physiological differences. Active standing, unlike passive head-up tilt, invokes the skeletal muscle pump. The European guidelines^10^ recommend active standing as the initial test for patients suspected of OH. However, the addition of high-quality heart rate and blood pressure recordings and other monitoring devices such as assessment of cerebral perfusion^32^ is cumbersome and thereby more difficult to achieve during active standing than during TT. Furthermore, with a diagnostic goal of inducing previously experienced symptoms, the duration of the upright period must be >20 min and, typically, 35 min.^10^ A long duration of active standing cannot be expected to be tolerated by many patients, especially the frail/aged.

Moving from supine to upright posture rarely induces syncope in normal healthy patients but may cause minor worrisome symptoms. For instance, a transient sensation of ‘greying out/dizziness/light-headedness/unsteadiness’ is common immediately after upright postural change (so-called ‘initial’ or ‘immediate’ OH). While usually harmless, this sensation may cause alarm/instability in some patients.^10^ Active standing is sufficient to document this problem and initiate treatment. Thus, active standing should be seen as a necessary, complementary aspect of cardiovascular autonomic workup in unexplained syncope, optimally with beat-to-beat haemodynamic monitoring for diagnostic accuracy.

Delayed OH is a far more important clinical problem, especially in older patients, debilitated patients, those with neurogenic OH, or in diseases that affect neurological responses, such as diabetes or alcohol abuse. In these cases, OH may be considerably delayed after change of posture that can result in fall injury. Active standing may not be tolerated for sufficient time to be diagnostic with additional fall injury risk during testing. TT avoids injury risk while providing the possibility of defining the diagnosis.

## Pathophysiology of syncope

Tilt testing has added greatly to our understanding of syncope mechanisms and different collapse patterns,^10^ but these are not further discussed in this review, which is focused on the clinical place of TT.

## Pros and cons of tilt testing

Since its clinical introduction, the utility of TT has not been without criticism, as discussed above in light of Kulkarni *et al*.’s^9^ opinion. It must be re-emphasized that TT is supported by evidence-based professional society practice guidelines.^10^ ^,^ ^11^ Furthermore, guidelines strongly emphasize that such testing is not necessary when the clinical history is clear-cut.^10^ ^,^ ^11^

Mortality is not an issue in most patients who undergo TT. The typical TT candidate is in a low-to-intermediate risk category, in whom a diagnosis is needed but has not been revealed at initial assessment by clinical history, physical examination including orthostatic blood pressure measurement and 12-lead ECG.^10^ ^,^ ^11^ ^,^ ^25–27^ TT is one means of reaching a diagnosis when not yet made; TT has almost no risks except rare, transient, atrial fibrillation, and very rare, prolonged, self-terminating asystole.^10^ TT demands detailed and thoughtful analysis of available data including that previously collected at initial clinical assessment where history from the patient and eyewitnesses of spontaneous syncope/collapse play the most important part.

Tilt-test reproducibility and estimated specificity and sensitivity are summarised in the recent European guidelines.^10^ It should be reiterated that there is no gold standard with which TT can be compared, although the follow-up expert review committee in FAST is a step towards.^25^ Reproducibility of positive tests is reduced in second tests and further in third tests to 80% positives in each.^15^ The decreasing positivity may be explained by the patient being aware of the unpleasantness of outcome, attempting in any way to avoid it. Leg movement is one obvious way. However, in severely affected VVS patients, reproducibility is high. This disadvantage of TT is rarely a clinical problem as repeat testing is seldom necessary.

False-positive outcomes occur with TT just as with any medical test, with a rate, expressed in terms of specificity and sensitivity,^5^ that is comparable with many widely used medical tests, such as exercise testing in daily cardiology practice. A positive TT in those who have never experienced syncope may be revealing a ‘hypotensive susceptibility’,^5^ which could manifest as syncope later in life. False negatives also occur but are over-ridden by the history. Analysis of the literature shows that TT has acceptable sensitivity and specificity,^10^ which should be distinguished from the positivity rate.^5^ ^,^ ^21^ It is, however, less good in the most difficult cases, which also applies widely in medicine.^5^ However, TT allows patients to confirm similarity, or its absence, of induced to spontaneous symptoms. The difficulty in some cases may be attributed to the overlap of a common reflex with another important condition, such as hypertrophic cardiomyopathy.

Tilt testing to monitor the effects of therapy is not recommended by European^10^ or North American^11^ practice guidelines. Nevertheless, TT can be useful, particularly in post-pacing syncope recurrence in severe VVS^33–38^ when combined with other cardiovascular autonomic tests such as carotid sinus massage.^10^ ^,^ ^36^ TT can help in pacemaker therapy selection^12^ and predict syncope recurrence after pacing; positive tilts pre-pacing are associated with a much higher recurrence rate than negative tilts with similarly positive ILR observations of VVS.^5^ ^,^ ^34–37^ An explanation may be that ‘hypotensive susceptibility’ is present even when dominated by cardioinhibition. Another may be that timing of development of hypotension ahead of bradycardia is very important^39^ but impossible to determine from ILR/insertable cardiac monitors (ICMs), which are yet unable to record blood pressure. TT may also have utility in reprogramming pacemakers after syncope recurrence, although this has not been widely adopted.^40^

Although TT may not be necessary to secure a diagnosis, it can serve to teach patients about prodromes so they can learn to invoke preventive measures, notably physical counter-measures, to abort subsequent episodes.^10^ ^,^ ^41^ Tilt provocation of symptoms can, thus, be an educational tool and is recommended by ESC guidelines as a class IIB indication.^10^

In the case of treatment selection, pacing using the closed-loop system offers stimulation earlier in the vasovagal reflex than awaiting bradycardia and, thus, a potentially effective therapy requiring consideration.^12^ ^,^ ^42^ ^,^ ^43^ However, if evidence from TT shows syncope due to hypotension preceding marked bradycardia by minutes, this may alter treatment strategy avoiding unnecessary pacemaker implantation.^39^ These issues have been reviewed in detail.^43^ Recent studies of TT methodology have provided greater insight into the sequence of haemodynamic events during VVS and may permit more appropriate application of pacing systems.^39^ ^,^ ^44^ ^,^ ^45^ The impact of reducing venous return and stroke volume during evolving VVS seems to be key to understanding the utility and limitations of pacing intervention.^44^

To summarize, TT has many pros in terms of its diagnostic, educational, patient reassurance and choice of pacing therapy with few cons other than being a lengthy procedure; importantly, it has virtually no risks. In contrast, active standing is really valuable only in immediate/classical OH. In delayed OH, it cannot replace tilt on grounds of haemodynamics and tolerability (*Table 1*).

## Cost containment and use of other testing

Management of syncope has become an unnecessarily expensive undertaking.^46^ Nevertheless, recent data indicate that up to 42% of patients admitted with syncope are discharged without a diagnosis and 23% are re-admitted for recurrences, often followed by non-diagnostic evaluation.^10^ ^,^ ^11^ The risk of adverse outcomes (e.g. mortality) is small but further expensive testing is not avoided. Many of these patients have undiagnosed VVS, which TT can provide preventing unnecessary and potentially harmful testing.

When the diagnosis is unclear from initial evaluation and there is no obvious cardiovascular cause for the episode, what is the next step? TT, when selected appropriately, following guidelines is cost-effective by avoiding use of more expensive and generally useless investigations, such as short-term ambulatory ECG monitoring (e.g. Holter monitoring), brain imaging, and EEG. European and North American guidelines are clear about this aspect of syncope investigation in recommending TT and advising against less effective, more expensive tests.^10^ ^,^ ^11^ Guidelines also suggest^10^ ^,^ ^11^ ^,^ ^28^ the use of TT as same-day assessment permitting diagnosis and preventing hospital admission.

Increasingly sophisticated, easy-to-place ILR/ICMs enhance diagnostic capacity but, even when chosen with care according to guideline recommendations,^10^ ^,^ ^11^ they may still not offer a definitive mechanistic cause of syncope. ILR/ICMs are expensive and delay diagnosis by awaiting symptom recurrence. Furthermore, blood pressure is not recorded during episodes, which is pertinent to vasodepressor responses. TT offers blood pressure and ECG recording albeit in a laboratory-induced, rather than spontaneous episode.^10^ However, ILR/ICMs are crucial for diagnosis when TT is negative or inconclusive, and patients suffer recurrences possibly with trauma. Thus, ILR/ICMs should be considered a necessary complement to holistic workup of unexplained syncope, not a TT competitor.

## Conclusion

Tilt testing is a useful and necessary diagnostic tool. Practice guidelines endorse its value based on published and strongly vetted evidence. TT adds importantly to our ability to appreciate the pathophysiology of syncope/collapse and improves care of our patients. Neither active standing nor ILR/ICMs can replace TT; active standing is valuable in some forms of OH but to date has no demonstrated value for other syncope presentations, while ILR/ICMs complement syncope workup.

**Conflict of interest:** R.S. is a consultant to Medtronic Inc., a member of speakers' bureau Abbott Laboratories Inc., and a stockholder in Edwards Lifesciences Corp. and Boston Scientific Inc. D.G.B. is supported in part by grant from the Dr Earl E Bakken Family in support of Heart-Brain research and reports consultant fees from Medtronic Inc. and Abbott Laboratories (SJM). A.F. reports personal fees from Biotronik Inc. and Medtronic Inc. H.A. is supported in part by research grants from Boston Scientific Inc., Medtronic Inc., and Abbott Laboratories. J.M.S. has funding from NHLBI Grant RO1HL134674. R.D.T. receives research support from Medtronic, consultancy fees from Theravance Biopharma, and lectures fees from Medtronic, Union Chimique Belge, and Novartis. B.O. reports personal fees from Amarin, Boehringer Ingelheim, Sanofi Aventis, Respicardia, and Lundbeck. J.G.v.D., M.B., F.d.L., P.B.L., R.A.K., A.M., S.D.R., and V.R. declare no conflicts of interest.
